# Clinicopathological features affecting the efficacy in ^131^I ablation therapy of papillary thyroid carcinoma with lymph node metastasis

**DOI:** 10.3389/fendo.2024.1382009

**Published:** 2024-07-17

**Authors:** Xiang Xu, Chengqian Li, Xiaolong Yu, Guoqiang Wang, Yanjun Guo, Huaiwen Ni, Wenjuan Zhao, Yangang Wang, Bingzi Dong

**Affiliations:** ^1^ Department of Geriatrics, The Affiliated Hospital of Qingdao University, Qingdao, China; ^2^ Department of Endocrinology and Metabolism, The Affiliated Hospital of Qingdao University, Qingdao, China; ^3^ Department of Nuclear Medicine, The Affiliated Hospital of Qingdao University, Qingdao, China; ^4^ Department of Endocrinology, Lanling County People’s Hospital of Linyi, Linyi, China

**Keywords:** Papillary thyroid carcinoma (PTC), lymph node metastasis, ^131^I ablation, excellent response (ER), cumulative risk

## Abstract

**Background:**

Lymph node metastasis is the major cause of increased recurrence and death in patients with papillary thyroid carcinoma (PTC). We evaluate the clinicopathologic factors affecting excellent response (ER) in patients with PTC with lymph node metastasis following operation and ^131^I ablation therapy.

**Methods:**

A total of 423 patients with PTC with lymph node metastasis who underwent thyroidectomy and postoperative ^131^I ablation therapy were enrolled. The relationship between clinicopathological factors affecting ER achievement was analyzed.

**Results:**

Multivariate analysis showed that the foci diameter (≤1 cm), unifocal, combination with Hashimoto’s thyroiditis (HT), lymph node metastases rate (LR) (≤40%), no postoperative lymph node metastasis, low preablative stimulated thyroglobulin (ps-Tg) level (≤3.87 ng/mL), and the time of ^131^I ablation therapy (one time) were positively correlated with the ER achievement [odds ratio (OR): 1.744, 3.114, 3.920, 4.018, 2.074, 9.767, and 49.491, respectively; all *p* < 0.05]. The receiver operating characteristic (ROC) curves showed that the cutoff values of ps-Tg and LR were 4.625 ng/mL and 50.50%, respectively. The AUC of ROC of ps-Tg and LR for predicting ER achievement was 0.821 and 0.746, respectively. The Tg and the cumulative risk of non-ER elevated with the increase of LR, especially for the high-level ps-Tg (>4.625 ng/mL) group.

**Conclusion:**

The foci diameter and number, combination with HT, LR, and ps-Tg level are independent factors for ER. Ps-Tg level and LR are valid predictive factors for the efficacy of ^131^I therapy in patients with PTC. The predictive value of the cumulative risk of non-ER can be improved by the combination of ps-Tg and LR.

## 1 Introduction

The incidence of papillary thyroid carcinoma (PTC) has increased over the past decades. The disease-related mortality is relatively low (<5%), but the recurrence rate reaches 30% (1). The rate of lymph node metastasis is approximately 20%–90% (1, 2), which is the major cause of recurrence and death in PTC (3). Relapse and prognosis of PTC depend on genetic and environmental interactions, including clinicopathological factors and genetic characteristics, particularly BRAF and TERT promoter mutations (4) and germline polymorphisms in the VEGF pathway (5). The synergistic prognostic effect between BRAF mutations and clinicopathological features has been identified. In addition, Marotta et al. also demonstrated that germline polymorphism of the VEGF pathway is a predictor of recurrence of non-advanced differentiated thyroid cancer (DTC) (4). Therefore, the 2015 American Thyroid Association (ATA) guidelines suggest a personalized non-categorical model including a wider range of variables to fit individual features (4, 6). The assessment of recurrence and prognosis of PTC relies on dynamic evaluation. Predictors with robust positive predictive value (PPV) are needed to elevate the PTC recurrence. Previously, the prediction of recurrence and mortality risk was mostly based on postoperative pathological features. However, because of the limitations of using pathological features alone, the ATA Guidelines for the first time proposed the response-to-therapy assessment system (RTAS) to assess the prognosis by monitoring thyroglobulin (Tg) and imaging examinations after ^131^I ablation (6). Studies suggest that the recurrence rate of patients with PTC with excellent response (ER) in the system is only 1%–4%, and the risk of tumor-related death is less than 1% (7), indicating that ER patients have a better prognosis.

Recent studies have shown that several clinicopathologic features may affect ER (8–10). However, those results are not consistent, and there is no clear optimal cutoff value as an indicator to predict ER and support treatment decision-making (8–10). Therefore, in this study, we investigated the potential impact of clinicopathologic features on ER after ^131^I ablation in patients with PTC with lymph node metastasis and evaluated the predictive value for ER and the cumulative risk of non-ER.

## 2 Participants and methods

### 2.1 Study design

A total of 423 patients with PTC who underwent total thyroidectomy and postoperative ^131^I ablation at the Affiliated Hospital of Qingdao University from January 2017 to October 2020 were enrolled. The inclusion criteria were as follows: (1) patients aged 20 to 80 years old who underwent total thyroidectomy, (2) postoperative pathological diagnosis confirmed as PTC, (3) patients received ^131^I radioiodine therapy at least one time, (4) no distant metastasis was confirmed by imaging and pathology, and (5) patients finished 2 years of follow-up. The participants with the following conditions were excluded: (1) pathologically confirmed as other types of thyroid carcinoma, (2) other underlying diseases including other malignant tumors or autoimmune disorders, and (3) positive thyroglobulin antibody (TgAb). Chest and abdominal computed tomography (CT) scan were performed before ^131^I ablation. The initial dosage of ^131^I ablation was set based on the recurrence risk stratification according to the 2015 ATA Guidelines (6). For low- and moderate-risk patients, the initial ^131^I ablation dose was 50–100 mCi. For high-risk patients with extraglandular invasion (larynx, trachea, esophagus, recurrent laryngeal nerve, striated muscle, etc.), the initial ^131^I ablation therapeutic dose was 120–180 mCi (6, 11). The patients achieved the goal of TSH > 30 mU/L after L-evothyroxine withdrawal and followed a low-iodine diet for 3–4 weeks. The patients were administrated L-T4 at day 3 after ^131^I ablation. The whole-body scan (Rx-WBS) and single-photon emission computed tomography/computed tomography (SPECT/CT) were performed within 1 week after ^131^I ablation (6).

During the 2-year follow-up, the first evaluation was performed at 3 months after initial ^131^I ablation therapy, and then dynamic evaluation was performed every 6 months during follow-up. Dynamic evaluation involved both serological and imaging measurements, including serum Tg levels, TgAb, thyroid function especially TSH level, and neck ultrasound. ^131^I diagnostic scanning (Dx-WBS), chest CT scan or ^18^F-FDG positron emission tomography (PET)-CT scanning, and fine needle aspiration (FNA) biopsy were also used if necessary.

Preablative stimulated thyroglobulin (ps-Tg) was defined as the Tg levels when TSH > 30 IU/mL as patients stopped taking L-T4 before ^131^I ablation (6). The diagnosis of Hashimoto’s thyroiditis (HT) was identified based on postoperative pathological analysis. Pre-operative lymph node metastasis was diagnosed by FNA (cytopathological diagnosis of PTC, or elevated Tg washout measurement) before surgery, or postoperative pathological analysis. Postoperative lymph node metastasis referred to the presence of metastatic lymph nodes detected by ^131^I-WBS or ultrasound and confirmed by FNA after surgery. Lymph node metastasis rate (LR) referred to the rate of involved lymph nodes, indicating the number of involved lymph nodes/total number of resected lymph nodes, based on postoperative pathological analysis.

### 2.2 Indication for thyroidectomy and lymph node dissection

In this study, patients with PTC with lymph node metastasis underwent total thyroidectomy according to the 2015 ATA Guidelines (6). Total thyroidectomy was performed for tumor diameter >4 cm. For the tumor diameter <4 cm (including diameter ≤1 cm), total thyroidectomy was performed under the following conditions: (1) bilateral foci; (2) lymph node metastasis in the lateral cervical area or lymph node metastasis number ≥5 or diameter ≥3 cm; (3) extracapsular invasion and metastatic lymph node invasion of surrounding tissues and organs, such as peripheral fat tissue, muscles, trachea, esophagus, laryngeal reentry nerve, and invaded blood vessels; and (4) high-risk factors such as head and neck radiotherapy history over the course of childhood and adolescence, and thyroid carcinoma family history.

The lymph node dissection was performed as follows: (1) central lymph node dissection: the therapeutic and prophylactic central-compartment lymph node dissection is routinely performed by experienced and skilled surgeons and the surrounding tissues were adequately protected; (2) lateral lymph node dissection: for PTC with lateral lymph node metastases confirmed by preoperative FNA or intraoperative frozen pathological examination, therapeutic lateral lymph node dissection was performed. The prophylactic lateral lymph node dissection is not routinely performed.

### 2.3 Efficacy evaluation criteria

According to the 2015 ATA Guidelines (6), the response to treatment was categorized into ER, indeterminate response (IDR), biochemical incomplete response (BIR), and structural incomplete response (SIR), based on Tg level and imaging examination. Based on the follow-up at 2 years, the recruited patients were divided into two groups, the ER group and the non-ER group. Patients with IR, BIR, and SIR were assigned as the non-ER group (6). Recurrence risk stratification and TNM staging were determined based on the 2015 ATA Guidelines (6) and the 8th edition TNM staging system of the American Joint Committee on Cancer (AJCC) (12).

### 2.4 Statistical analysis

Statistical analysis was performed using the SPSS 26.0 software. Categorical variables were presented as frequencies and percentages, and two-group comparison was performed by the *χ* ^2^ test. Partial distributed data were presented as the median value and comparisons between two groups were performed using the Mann–Whitney *U* test. Logistic regression analysis was used to analyze the factors affecting the achievement of ER. Prognostic receiver operating characteristic (ROC) curves were applied to obtain the optimal threshold for estimating ER achievement, and the prediction of the cumulative risk of non-ER was performed using the Kaplan–Meier model. The statistical significance was *p* < 0.05 compared between two groups. The comparison among the three groups and four groups was *p* < 0.017 and *p* < 0.008, which were considered statistically significant, respectively.

## 3 Results

### 3.1 Clinicopathologic features of patients with PTC

A total of 423 patients with PTC with lymph node metastasis were enrolled, with age ranging from 20 to 78 years (average age, 43.82 ± 11.85 years old). The male-to-female ratio was 1:2.18. The follow-up time was 24–32 months. The maximum diameter of tumor foci was 0.1–5.5 cm, averaging 1.57 ± 1.06 cm. The number of foci was one to eight (average foci number was 1.81 ± 1.09). The number of lymph node metastasis was 1–36, with a median of 4. Lymph node metastasis rate (% of involved metastatic lymph nodes/total number of resected lymph nodes) was 5%–100%, with a median of 40%. There were 226 cases (53.43%) with extracapsular invasion, including 146 cases (34.52%) in the strap muscles, 44 cases (10.43%) with recurrent laryngeal nerve invasion, 18 cases (4.25%) with tracheal invasion, 6 cases (1.41%) with esophageal invasion, 6 cases (1.41%) with blood vessel (internal jugular vein, common carotid artery, and transverse cervical artery) invasion, and 6 cases (1.41%) with surrounding soft tissue invasion. The clinicopathologic features are shown in **Table 1**.

**Table 1:** Clinicopathologic data of enrolled patients with PTC.

Clinical indicators, *n* (%)	Clinical indicators, *n* (%)
Age (years) <55 341 (80.61%) ≥55 82 (19.39%) Sex Male 133 (31.44%) Female 290 (68.56%) Number of primary foci Unifocal 221 (52.25%) Multifocal 202 (47.75%) Unilateral/Bilateral foci of primary tumor Unilateral 265 (62.25%) Bilateral 158 (37.75%) Maximum diameter of primary foci (cm) ≤1 176 (41.61%) >1 247 (58.39%) Extracapsular invasion Yes 226 (53.43%) No 197 (46.57%) Combination with HT Yes 71 (16.78%) No 352 (83.22%) Laterality of lymph node metastasis Ipsilateral 305 (72.10%) Bilateral 118 (27.90%) Ps-Tg (ng/mL) ≤3.87 213 (50.35%) >3.87 210 (49.56%) Number of lymph node metastases # <5 218 (51.54%) ≥5 205 (48.46%)	Lymph node metastasis rate # ≤40% 209 (49.41%) >40% 214 (50.59%) Postoperative lymph node metastasis No 351 (82.97%) Yes 72 (17.03%) Metastatic lymph node region Central only 51 (12.06%) Lateral only 43 (10.16%) Central+lateral 329 (77.78%) Initial dose of ^131^I therapy (mCi) ≤100 (50–100) 270 (63.83%) >100 (120–180) 153 (36.17%) Frequency of ^131^I therapy 1 time 357 (84.40%) 2 times 51 (12.06%) ≥3 times (3–4) 15 (3.54%) Total dose of ^131^I therapy (mCi) 50–100 250 (59.10%) 120–150 112 (26.48%) 160–200 5 (1.18%) >200 (220–600) 56 (13.24%) Recurrence risk stratification Low risk 33 (7.8%) Intermediate risk 184 (43.49%) High risk 206 (48.71%) TNM staging Phase I 344 (81.32%) Phase II 53 (12.53%) Phase III 20 (4.73%) Phase IV 6 (1.42%)

# Presented as median. PTC, papillary thyroid cancer; HT, Hashimoto’s thyroiditis.

### 3.2 Comparison of clinicopathologic features between ER and non-ER groups

Based on the response to ^131^I ablation after 2 years of follow-up, 314 patients with PTC achieved ER with an ER rate of 74.2%. The patients with PTC were divided into ER and non-ER groups. The results showed that the proportion of female patients, unifocal, primary foci ≤1 cm in diameter, no extracapsular invasion, combination with HT, low ps-Tg level (≤3.87 ng/mL), and low risk of recurrence risk stratification were significantly higher in the ER group than in the non-ER group (all *p* < 0.05). However, age, laterality of tumor foci, and TNM staging did not show significant difference between two groups (all *p* > 0.05) (**Table 2**).

**Table 2:** Comparison of clinicopathologic features between ER and non-ER groups at 2 years follow-up (*n*, %).

		ER (*n* = 314, 74.23%)	Non-ER (*n* = 109, 25.77%)	*χ* ^2^	p
Sex	Male	93 (29.62)	40 (36.70)	4.529	0.033
	Female	221 (70.38)	69 (63.30)		
Age (years)	<55	248 (78.98)	92 (84.40)	1.600	0.209
	≥55	66 (21.02)	17 (15.60)		
Number of primary tumor foci	Unifocal	176 (56.05)	48 (44.04)	4.688	0.03
	Multifocal	138 (43.95)	61 (55.96)		
Laterality of primary tumor foci	Unilateral	200 (63.69)	64 (58.72)	0.737	0.391
	Bilateral	114 (36.31)	45 (41.28)		
Maximum diameter of primary tumor foci (cm)	≤1	141 (44.90)	35 (32.11)	5.764	0.017
	>1	173 (55.10)	74 (67.89)		
Extracapsular invasion	Yes	158 (50.31)	68 (62.38)	4.735	0.030
	No	156 (49.69)	41 (37.62)		
Combination with HT	Yes	64 (20.38)	7 (6.42)	11.483	0.001
	No	250 (79.62)	102 (93.58)		
ps-Tg (ng/mL) #	≤3.87	198 (63.06)	15 (13.76)	76.945	<0.001
	>3.87	116 (36.94)	94 (86.24)		
Recurrence risk stratification	Low risk	30 (9.55)	3 (2.75)	4.302	0.038
	Medium–high risk	284 (90.45)	106 (97.25)		
TNM staging	Phase I/II	297 (94.6%)	100 (91.7%)	1.134	0.287
	Phase III/IV	17(5.4%)	9 (8.3%)		

# ps-Tg value was not normally distributed and was presented as median. ER, excellent response; HT, Hashimoto’s thyroiditis.

Comparison of the pathologic characteristics of metastatic lymph nodes between ER and non-ER groups was analyzed. The ER group had a lower number and rate of lymph node metastases, less postoperative lymph node metastases, and a significantly lower proportion of lymph nodes located in the central and cervical lateral region than the non-ER group (all *p* < 0.05) (**Table 3**).

**Table 3:** Comparison of pathologic features of metastatic lymph nodes between ER and non-ER groups (*n*, %).

Groups		ER (*n* = 314, 4.23%)	Non-ER (*n* = 109, 5.77%)	*χ* ^2^	p
Number of lymph node metastases #	<5	177 (56.37)	41 (37.61)	11.395	0.01
	≥5	137 (43.63)	68 (62.39)		
Lymph node metastasis rate #	≤40%	169 (53.82)	41 (37.61)	8.502	0.004
	>40%	145 (46.18)	68 (62.39)		
Laterality metastasis of lymph node	Ipsilateral	236 (75.16)	69 (63.30)	5.655	0.017
	Bilateral	78 (24.84)	40 (36.70)		
Metastatic lymph node region	Central	40 (12.73)	10 (9.17)	2.07^a^, 1.71^b^, 7.46^c^	0.150^a^, 0.191^b^, 0.006^c^
	Lateral	39 (12.42)	4 (3.67)		
	Central+lateral	235 (74.85)	95 (87.16)		
Postoperative lymph node metastasis	Yes	46 (14.64)	26 (28.85)	4.853	0.028
	No	268 (85.46)	83 (71.15)		

#Grouped by median. a indicates the comparison between the central group and the lateral group. b indicates the comparison between the central group and the central+lateral group. c indicates the comparison between the lateral group and the central+lateral group. Statistically significant differences were considered as p < 0.017.

In this study, the ER group had an increased rate of HT. Therefore, we further analyzed the relationship between HT and clinicopathological features. The results showed that female patients (female patients 84.50% vs. male patients 15.50%) and non-extracapsular invasion (non-extracapsular invasion 61.97% vs. extracapsular invasion 38.03%) in the HT group (71 cases, 16.78%) were significantly higher than those in the non-HT group (352 cases, 83.22%) (all *p* < 0.05), while age, number of primary tumor foci, the maximum diameter of primary foci, unilateral/bilateral foci, number of lymph node metastases, LR, and postoperative lymph node metastasis showed no difference between HT and non-HT groups.

To analyze the relationship between frequency/dosage of ^131^I ablation and ER, we found that the ER rate in the 1 time ^131^I ablation therapy group was higher than that in the 2 times and ≥3 times ^131^I ablation therapy group (all *p* < 0.017). In addition, the ER rate in the low initial ^131^I ablation dose ≤100 (50–100) mCi group was significantly higher than that in the high-dose >100 (120–180) mCi group (*p* < 0.05). We further divided the patients into four groups based on the total dose of ^131^I therapy. The results showed that the ER rate of the ^131^I therapy total dose 50–100 mCi group was significantly higher than that of the 120–150 mCi group, as well as the >200–(220–600) mCi group (all *p* < 0.008) (**Table 4**).

**Table 4:** Comparison of times and dosage of ^131^I ablation therapy between ER and non-ER groups in patients with PTC.

		ER (*n* = 314, 74.23%)	Non-ER (*n* = 109, 25.77%)	*χ* ^2^	p
Frequency of ^131^I therapy	1 time	291 (85.34)	65 (59.63)	46.172^a^ 32.869^b^ 1.760^c^	<0.001^a^ <0.001^b^ 0.185
	2 times	20 (6.37)	32 (29.36)		
	≥3 times	3 (8.29)	12 (11.01)		
Initial dose of ^131^I therapy (mCi)	≤100 (50–100)	221 (70.38)	49 (44.95)	23.511	<0.001
	>100 (120–180)	93 (29.62)	60 (55.05)		
Total dose of ^131^I therapy (mCi)	50–100	211 (67.19)	39 (35.78)	10.290^d^, 0.074^e^, 58.723^f^, 0.234^g^ 18.173^h^, 3.864^i^	0.001^d^, 0.786^e^, <0.001^f^, 0.629^g^ ^<^0.001^h^, 0.049^i^
	120–150	79 (25.15)	33 (30.27)		
	160–200	4 (1.27)	1 (0.92)		
	>200 (220–600)	20 (6.39)	36 (32.13)		

a and b indicate the comparison of the ^131^I ablation therapy 1 time group with the 2 times group and the ≥3 times group, respectively. c indicates the comparison of the 2 times group with the ≥3 times group. p < 0.017 was defined as statistically significant.

d, e, and f indicate the comparison of the total dose of the 50–100 group with the 120–150, 160–200, and >200 (220–600) (mCi) groups, respectively.

g and h indicate the comparison of the total dose of the 120–150 group compared with the 160–200 and >200 (220–600) (mCi) groups, respectively.

i indicates the total dose of the 160–200 group compared with the >200 (220–600) (mCi) group, and the difference was considered statistically significant at p < 0.008.

### 3.3 The independent risk factors affecting ER achievement after ^131^I therapy

Multivariate logistic regression analysis was performed to analyze the factors associated with ER after ^131^I therapy. The results revealed that the maximum diameter of tumor foci (≤1 cm), unifocal, combination with HT, lymph node metastases rate (≤40%), no postoperative lymph node metastasis, lower level of ps-Tg (≤3.87 ng/mL), and the frequency of ^131^I therapy (one time) were independent risk factors that positively correlated with the ER achievement (OR: 1.744, 3.114, 3.920, 4.018, 2.074, 9.767, and 49.491, respectively; all *p* < 0.05). There was no correlation between ER and the number of metastatic lymph nodes, laterality of lymph node metastasis, extracapsular invasion, or initial dose and total dose of ^131^I therapy (all *p* > 0.05) (**Table 5**).

**Table 5:** Multi-factorial analysis of clinicopathological features affecting therapy response to ^131^I ablation therapy.

Clinicopathological features (1, 0; analyzed variable 1)	OR	95% CI	p
Maximum diameter of tumor foci (≤1 cm, >1 cm)	1.744	1.030–2.951	0.038
Number of primary tumor foci (unifocal/multifocal)	3.114	1.364–7.109	0.007
Combination with HT (yes, no)	3.920	1.646–9.332	0.002
Extracapsular invasion (no, yes)	0.661	0.400–1.091	0.105
Number of metastatic lymph nodes (<5, ≥5)	1.429	0.810–2.522	0.218
Lymph node metastases rate (≤40%, >40%)	4.018	2.351–6.867	0.000
Metastatic lymph node region (central, lateral, central+lateral)	0.902	0.368–2.210	0.822#
Laterality of metastatic lymph nodes (ipsilateral, bilateral)	0.579	0.129–2.612	0.478
Postoperative lymph node metastasis (no, yes)	2.074	1.090–3.3.948	0.026
Ps-Tg values (≤3.87, >3.87)	9.767	5.171–18.448	0.000
Frequency of ^131^I therapy (1, 2, ≥3 times)	49.491	3.864–633.817	0.003##
Initial dose of ^131^I ablation therapy [≤ 100 (50–100), >100 (120–180) mCi]	3.274	0.752–14.257	0.114
Total dose of ^131^I ablation therapy [50–100, 120–150, 160–200, >200 (220–600) mCi]	0.227	0.019–2.752	0.244

# indicates the comparison between metastatic lymph nodes located in the lateral region and those in the central+lateral region; ## indicates the number of 1 time versus ≥3 times ^131^I therapy.

### 3.4 Predictive value of ps-Tg and LR in ER achievement

**Figure 1:**
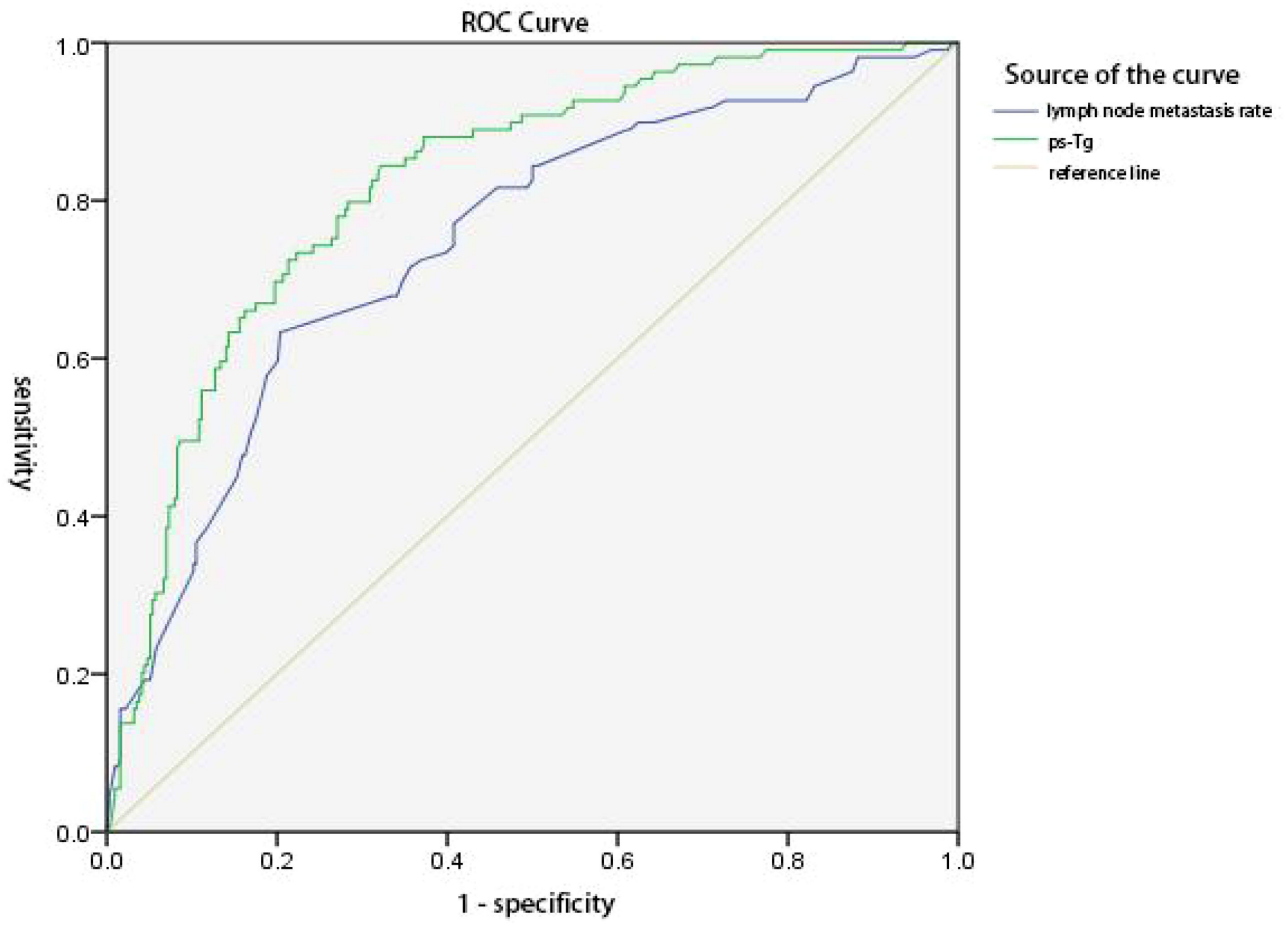
ROC curves of ps-Tg values and lymph node metastasis rate (LR) for predicting the excellent response (ER). The area under the curve (AUC) was 0.821 (95% CI 0.777–0.865) and 0.746 (95% CI 0.691–0.800), respectively. The Youden index was 0.522 and 0.429, respectively. The best cutoff values were 4.625 ng/mL for ps-Tg and 50.50% for LR, with a corresponding sensitivity of 84.4% and 63.3%, a specificity of 67.8% and 79.6%, a positive predictive value of 92.6% and 86.2%, and a negative predictive value of 48.7% and 51.8%, respectively.

The predictive value of ps-Tg level and LR in ER achievement was analyzed, respectively. The results showed that the maximum area under the curve (AUC) of the ROC curve was 0.821 (95% CI 0.777–0.865) and 0.746 (95% CI 0.691–0.800). The best cutoff values were 4.625 ng/mL for ps-Tg and 50.50% for LR, with corresponding sensitivities of 84.4% and 63.3%, specificities of 67.8% and 79.6%, PPVs of 92.6% and 86.2%, and negative predictive values of 48.7% and 51.8%, respectively. Results indicate that ps-Tg ≤ 4.625 ng/mL and LR ≤ 50.50% are effective factors for predicting ER achievement (**Figure 1**).

**Figure 2:**
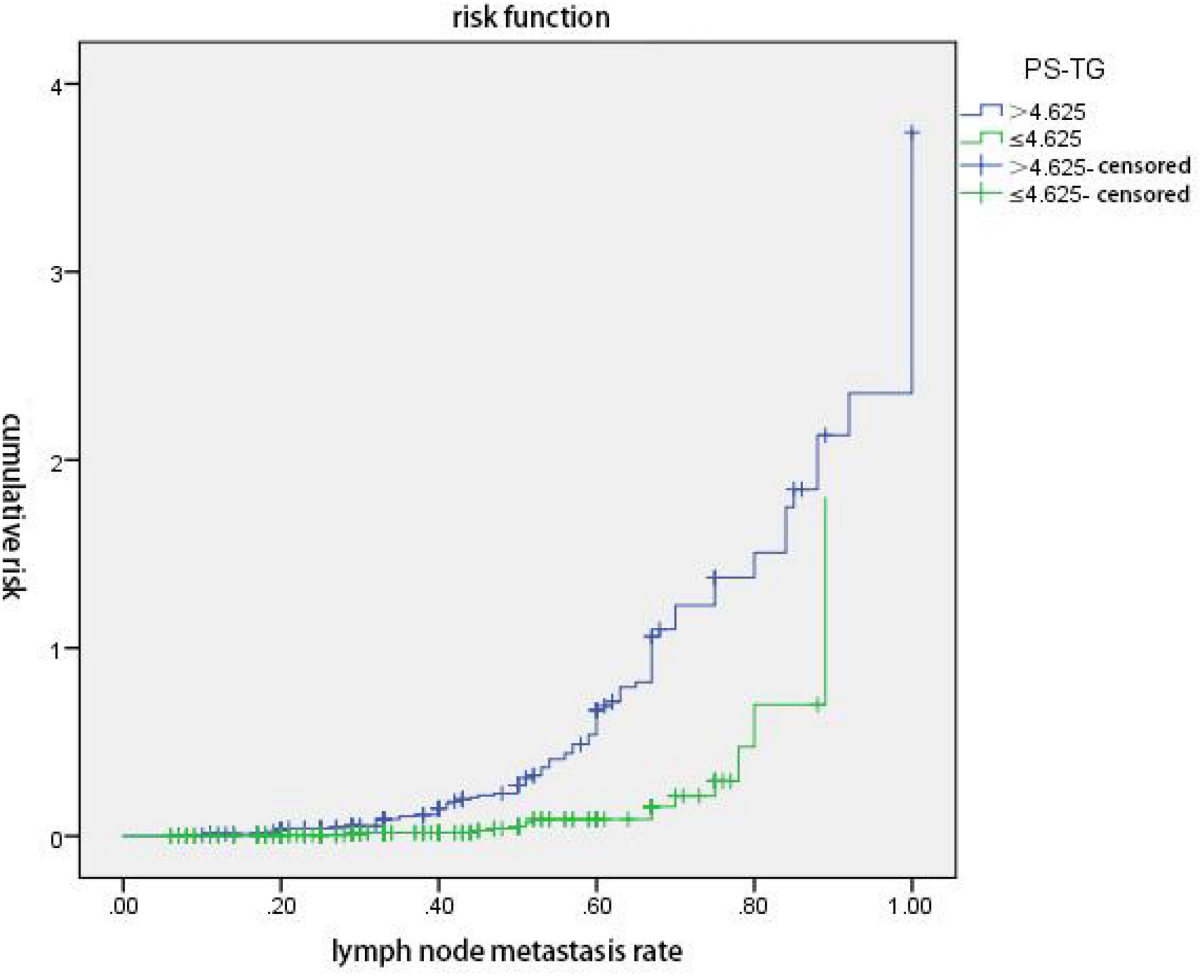
ps-Tg combined with lymph node metastasis rate (LR) predicts the cumulative risk of non-excellent response (non-ER). Regardless of whether the ps-Tg value was at low level (≤4.625 ng/mL) or high level (>4.625 ng/mL), the cumulative risk of non-ER elevated with the increase of LR, especially for the high-level ps-Tg group. At the same LR, the cumulative risk of non-ER was higher in the high-level ps-Tg group (*p* < 0.05).

The ps-Tg value and LR were analyzed for the joint prediction of the cumulative risk for non-ER. The results revealed that regardless of whether the ps-Tg value was at low level (≤4.625 ng/mL) or high level (>4.625 ng/mL), the cumulative risk of non-ER elevated with the increase of LR, especially for the high-level ps-Tg group, whereas at the same LR, the cumulative risk of non-ER was higher in the high-level ps-Tg group than the low-level ps-Tg group (both *p* < 0.05) (**Figure 2**).

## 4 Discussion

Although most patients with PTC have a good prognosis with low mortality and long survival, the recurrence rate can be up to 30% due to the presence of lymph node metastasis (13–15). Therefore, active and rationalized treatment strategies and dynamic follow-up for assessment of PTC with lymph node metastasis are important.

^131^I ablation therapy as an adjuvant treatment for PTC with lymph node metastasis has been widely applied. Several studies have reported that the effectiveness of ^131^I ablation therapy in patients with PTC with lymph node metastasis can reach up to 71.4%–88.07% (16, 17). Previously, Gao et al. analyzed the data from low- and intermediate-risk patients with PTC and showed an ER rate of 93.7% and 78.2% after ^131^I treatment (18), while Zhao et al. showed a 54.5%–73.2% ER achievement, which differed from high–low TSH level stratification (19). In our study, we found that the overall ER rate was 74.2%, which was comparable to the results of previous studies.

In recent years, several studies revealed the factors influencing the clinical outcome after postoperative ^131^I ablation in patients with PTC. The ps-Tg level has been proven to be the predictor of recurrence risk and prognosis (20, 21). Li et al. (8) reported that low ps-Tg level was more likely to achieve ER in moderate-risk patients with PTC. Wang et al. (9) also concluded that a higher level of ps-Tg was associated with a lower ER rate of the initial ^131^I ablation. However, there were controversies regarding the relationship between ER and the diameter and number of tumor foci, extracapsular invasion, and the number and rate of lymph node metastasis. Several studies revealed that tumor size, number of lymph nodes, LR, and lymph node size were significantly associated with ER achievement after ^131^I ablation therapy (8, 9, 22). However, Shangguan et al. (23) reported that the LR did not correlate with ER. In this study, we investigated the relationship between clinicopathological factors and ER. The results showed that tumor diameter (≤1 cm), unifocal, combination with HT, low lymph node metastases rate (≤40%), the absence of postoperative lymph node metastasis, and the low level of ps-Tg (≤3.87 ng/mL) were independent factors positively correlated with the ER achievement.

The ROC of ps-Tg and LR showed high sensitivity and PPV for ER achievement. Based on the significance of ps-Tg in predicting ER and recurrence or metastasis (20, 24, 25), ps-Tg level was used as an important serological indicator in the evaluation system in guiding the individualized follow-up and treatment of patients with PTC. However, there is variability in the thresholds of ps-Tg level as indicators in different studies, which may be related to the different clinicopathologic characteristics of the selected patients, the area of operation, the dosage and frequency of ^131^I ablation, and the criteria for evaluating the efficacy, the follow-up frequency.

In clinical practice, patients with PTC usually exhibit two or more malignant pathologic features at the same time, which undoubtedly aggravates the progression and reduces the likelihood of achieving ER. We revealed that serum ps-Tg and lymph node metastasis rate are important indicators to predict ER achievement in this study, and further results showed that the cumulative risk of non-ER elevated with the increase of lymph node metastasis rate, regardless of ps-Tg level (low level ≤4.625 ng/mL or high level >4.625 ng/mL). Moreover, the increase of cumulative risk was more prominent in the high ps-Tg level group. Hence, the prediction of non-ER achievement can be improved by a combination of evaluating ps-Tg and LR, which can avoid the limitation of assessment by a single factor. A combination of ps-Tg and LR could predict the efficacy of ^131^I ablation in advance, potentially useful for individualized therapeutic assessment.

Regarding the studies on the relationship between HT and PTC, HT is associated with the development of PTC and is widely recognized as a pre-disease state of PTC (26). However, the effect of HT on the pathological features of PTC is inconsistent (27, 28). Studies suggested that HT is a “double-edged sword” in patients with PTC, which increases the risk of PTC but is a protective factor against progression (29), and lymphocyte infiltration and cytokines derived from lymphocytes may attenuate tumor invasiveness and proliferation (27, 28). In contrast, some studies indicated that HT promotes PTC development progression, which is associated with both the endocrine mechanism by promoting TSH increasing and immune mechanism via the reduced expression of major histocompatibility complex (MHC)-I molecules, leading to the upregulation of immunosuppressive components and immune escape (30, 31). Several studies showed that there was no correlation between combined HT and ER (32, 33), while a report from Lim et al. suggested that patients with PTC combined with HT had a low ER rate after ^131^I ablation (34). A large-scale prospective multicenter study analyzed the relationship between autoimmune thyroiditis and DTC outcomes. The results showed that patients with autoimmune thyroiditis were more frequently categorized as low and intermediate risk. The biochemical persistence was more frequent in autoimmune thyroiditis patients, but no association between AT and structural persistence of disease. Patients with autoimmune thyroiditis had a more frequently indeterminate response. These findings may be explained by the presence of a residual thyroid tissue (35). Of note, our data differed from the above finding, showing that combination with HT is an independent factor of ER achievement, and a higher proportion of patients have an absence of extracapsular extension in the combination with the HT group (61.97% vs. 38.03%) and are positively correlated with ER, suggesting that PTC combined with HT is less invasive, which may contribute to ER achievement. The variability of the findings in different studies may be related to several factors, such as regional differences in the study populations, varying iodine nutritional status, and different genetic backgrounds.

In addition, our study revealed that less time of ^131^I ablation therapy (only one time) was correlated with ER outcomes, indicating that patients with mild disease have a better outcome. We also found a higher proportion of low risk of recurrence in the ER group, suggesting that the frequency and intensity of follow-up could be reduced.

We acknowledge some limitations of our study. Since the patients were from a single medical center and retrospective study, selection bias could not be ruled out. Second, we cannot exclude the influence of lifestyle (such as iodine content in diet and smoking) or hereditary factors of the enrolled patients. Third, considering the inert nature of PTC, further studies with a long-term follow-up are needed.

In summary, we have found that the diameter of tumor foci ≤1 cm, unifocal, combination with HT, the absence of postoperative lymph node metastasis, lower LR, and a lower level of ps-Tg were independent factors correlated with the ER achievement. The ps-Tg and LR had a predictive value for ER achievement. The predictive value of the cumulative risk of non-ER can be improved by a combination of evaluating ps-Tg and LR.

## Data availability statement

The original contributions presented in the study are included in the article/supplementary material. Further inquiries can be directed to the corresponding authors.

## Ethics statement

The ethics committee of the Affiliated Hospital of Qingdao University approved this study. The studies were conducted in accordance with the local legislation and institutional requirements. The participants provided their written informed consent to participate in this study.

## Author contributions

XX: Formal analysis, Investigation, Writing – original draft. CL: Formal analysis, Investigation, Project administration, Writing – original draft. XY: Investigation, Methodology, Writing – original draft. GW: Methodology, Writing – original draft. YG: Data curation, Formal analysis, Methodology, Writing – original draft. HN: Data curation, Investigation, Writing – original draft. WZ: Conceptualization, Supervision, Writing – review & editing. YW: Conceptualization, Resources, Supervision, Writing – review & editing. BD: Conceptualization, Funding acquisition, Investigation, Project administration, Supervision, Visualization, Writing – original draft, Writing – review & editing.

## References

[1] Shaha AR. Prognostic factors in papillary thyroid carcinoma and implications of large nodal metastasis. Surgery. (2004) 135:237. doi: 10.1016/j.surg.2003.08.023 14739864

[2] Chow SM Law SC Chan JK Au SK Yau S Lau WH. Papillary microcarcinoma of the thyroid—Prognostic significance of lymph node metastasis and multifocality. Cancer. (2003) 98:31–40. doi: 10.1002/cncr.11442 12833452

[3] Vaisman F Tala H Grewal R Tuttle RM. In differentiated thyroid cancer, an incomplete structural response to therapy is associated with significantly worse clinical outcomes than only an incomplete thyroglobulin response. Thyroid. (2011) 21:1317–22. doi: 10.1089/thy.2011.0232 22136267

[4] Marotta V Sciammarella C Colao A Faggiano A. Application of molecular biology of differentiated thyroid cancer for clinical prognostication. Endocr Relat Cancer. (2016) 23:R499–515. doi: 10.1530/ERC-16-0372 27578827

[5] Marotta V Sciammarella C Capasso M Testori A Pivonello C Chiofalo MG. Germline polymorphisms of the VEGF pathway predict recurrence in nonadvanced differentiated thyroid cancer. J Clin Endocrinol Metab. (2017) 102:661–71. doi: 10.1210/jc.2016-2555 27849428

[6] Haugen BR Alexander EK Keith CB Doherty GM Mandel SJ Nikiforov YE. 2015 American thyroid association management guidelines for adult patients with thyroid nodules and differentiated thyroid cancer: the American thyroid association guidelines task force on thyroid nodules and differentiated thyroid cancer. Thyroid. (2016) 26:1–133. doi: 10.1089/thy.2015.0020 26462967 PMC4739132

[7] Tuttle RM Alzahrani AS. Risk stratification in differentiated thyroid cancer: from detection to final follow-up. J Clin Endocrinol Metab. (2019) 104:4087–100. doi: 10.1210/jc.2019-00177 PMC6684308 30874735

[8] Li YX Rao MH Zheng CX Huang J Fang D Xiong Y. Analysis of factors influencing the clinical outcome after surgery and 131I therapy in patients with moderate-risk thyroid papillary carcinoma. Front Endocrinol (Lausanne). (2022) 13:1015798. doi: 10.3389/fendo.2022.1015798 36313750 PMC9613939

[9] Wang C Diao HC Ren P Wang X Wang Y Zhao W. Efficacy and affecting factors of 131I thyroid remnant ablation after surgical treatment of differentiated thyroid carcinoma. Front Oncol. (2018) 8:640. doi: 10.3389/fonc.2018.00640 30619772 PMC6306449

[10] Ju NT Hou LY Song HJ Qiu Z Wang Y Sun Z. TSH ≥30 mU/L may not be necessary for successful 131I remnant ablation in patients with differentiated thyroid cancer. Eur Thyroid J. (2023) 12:e220219. doi: 10.1530/ETJ-22-0219 37022724 PMC10305696

[11] Cooper DS Doherty GM Haugen BR Kloos RT Lee SL Mandel SJ. Revised American thyroid association management guidelines for patients with thyroid nodules and differentiated thyroid cancer. Thyroid. (2009) 19:1167–214. doi: 10.1089/thy.2009.0110 19860577

[12] Tuttle RM Haugen B Perrier ND. Updated American joint committee on cancer/tumor-node-metastasis staging system for differentiated and anaplastic thyroid cancer (Eighth edition): what changed and why? Thyroid. (2017) 27:751–6. doi: 10.1089/thy.2017.0102 PMC5467103 28463585

[13] Hundahl SA Phillips JL Menck HR. The National Cancer Data Base report on poor survival of U.S. gastric carcinoma patients treated with gastrectomy: Fifth edition American Joint Committee on Cancer staging, proximal disease, and the “different disease” hypothesis. Cancer. (2000) 88:921–32. doi: 10.1002/(ISSN)1097-0142 10679663

[14] Rusinek D Chmielik E Krajewska J Jarzab M Oczko-Wojciechowska M Czarniecka A. Current advances in thyroid cancer management. Are we ready for the epidemic rise of diagnoses? Int J Mol Sci. (2017) 18:1817. doi: 10.3390/ijms18081817 28829399 PMC5578203

[15] Scheffel RS Zanella AB Antunes D Dora JM Maia AL. Low recurrence rates in a cohort of differentiated thyroid carcinoma patients: A referral center experience. Thyroid. (2015) 25:883–9. doi: 10.1089/thy.2015.0077 26061907

[16] He Y Pan MZ Huang JM. Iodine-131: an effective method for treating lymph node metastases of differentiated thyroid cancer. Med Sci Monitor Int Med J Exp Clin Res. (2016) 22:4924–8. doi: 10.12659/MSM.899028 PMC5181522 27974741

[17] Yang Y Gan M Yi K Han S Lin Z Shi Y. Guiding the postoperative radioactive iodine-131 therapy for patients with papillary thyroid carcinoma according to the prognostic risk groups: a SEER-based study. J Cancer Res Clin Oncol. (2023) 149:17147–57. doi: 10.21203/rs.3.rs-2835496/v1 PMC11796916 37782329

[18] Gao HY Huang JY Qing J Dai QJ. Radioiodine (131I) treatment decision-making for low- and intermediate-risk differentiated thyroid cancer. Arch Endocrinol Metab. (2023) 67:197–205. doi: 10.20945/2359-3997000000538 36651706 PMC10689029

[19] Zhao T Liang J Guo Z Li T Lin Y. In patients with low- to intermediate-risk thyroid cancer, a preablative thyrotropin level of 30 μIU/mL is not adequate to achieve better response to 131I therapy. Clin Nucl Med. (2016) 41:454–8. doi: 10.1097/RLU.0000000000001167 26914559

[20] Yang X Liang J Li TJ Yang K Liang DQ Yu Z. Postoperative stimulated thyroglobulin level and recurrence risk stratification in differentiated thyroid cancer. Chin Med J (Engl). (2015) 128:1058–64. doi: 10.4103/0366-6999.155086 PMC4832946 25881600

[21] Gonzalez C Aulinas A Colom C Tundidor D Mendoza L Corcoy R. Thyroglobulin as early prognostic marker topredict remission at 18–24 months in differentiated thyroid carcinoma. Clin Endocrinol (Oxf). (2014) 80:301–6. doi: 10.1111/cen.12282 23826916

[22] Yin Y Xu X Shen L Zhao W Diao H Li C. Influencing factors and cumulative risk analysis of cervical lymph node metastasis of papillary thyroid microcarcinoma. Front Oncol. (2021) 11:644645. doi: 10.3389/fonc.2021.644645 34660255 PMC8514816

[23] Shangguan L Fang S Zhang P Han S Shen X Geng Y. Impact factors for the outcome of the first 131I radiotherapy in patients with papillary thyroid carcinoma after total thyroidectomy. Ann Nucl Med. (2018) 33:177–83. doi: 10.1007/s12149-018-01321-w 30515649

[24] Szujo S Bajnok L Bodis B Nagy Z Nemes O Rucz K. The prognostic role of postablative non-stimulated thyroglobulin in differentiated thyroid cancer. Cancers (Basel). (2021) 13:310. doi: 10.3390/cancers13020310 33467717 PMC7830405

[25] Couto JS Almeida MFO Trindade VCG Marone MMS Scalissi NM Cury AN. A cutoff thyroglobulin value suggestive of distant metastases in differentiated thyroid cancer patients. Braz J Med Biol Res. (2020) 53:e9781. doi: 10.1590/1414-431x20209781 33053096 PMC7561073

[26] Lee JH Kim Y Choi JW Kim YS. The association between papillary thyroid carcinoma and histologically proven Hashimoto's thyroiditis: a meta-analysis. Eur J Endocrinol. (2013) 168:343–9. doi: 10.1530/EJE-12-0903 23211578

[27] Mazokopakis EE Tzortzinis AA Dalieraki-Ott EI Tsartsalis AN Syros PK Karefilakis CM. Coexistence of Hashimoto's thyroiditis with papillary thyroid carcinoma. A retrospective study. Hormones (Athens Greece). (2010) 9:312–7. doi: 10.14310/horm.2002.1149 21112862

[28] Del R Cataldo S Sommaruga L Concione L Arcuri MF Sianesi M. The association between papillary carcinoma and chronic lymphocytic thyroiditis: does it modify the prognosis of cancer? Minerva Endocrinol. (2008) 33:1–5. 18277374

[29] Xu J Ding K Mu L Huang J Ye F Peng Y. Hashimoto's thyroiditis: A "Double-edged sword" in thyroid carcinoma. Front Endocrinol (Lausanne). (2022) 13:801925. doi: 10.3389/fendo.2022.801925 35282434 PMC8907134

[30] Han LT Hu JQ Ma B Wen D Zhang TT Lu ZW. IL-17A increases MHC class I expression and promotes T cell activation in papillary thyroid cancer patients with coexistent Hashimoto's thyroiditis. Diagn Pathol. (2019) 14:52. doi: 10.1186/s13000-019-0832-2 31159823 PMC6547553

[31] Wang T Shi J Li L Zhou X Zhang H Zhang X. Single-cell transcriptome analysis reveals inter-tumor heterogeneity in bilateral papillary thyroid carcinoma. Front Immunol. (2022) 13:840811. doi: 10.3389/fimmu.2022.840811 35515000 PMC9065345

[32] Carvalho MS Rosario PW Mourão GF Calsolari MR. Chronic lymphocytic thyroiditis does not influence the risk of recurrence in patients with papillary thyroid carcinoma and excellent response to initial therapy. Endocrine. (2017) 55:954–8. doi: 10.1007/s12020-016-1185-1 27878772

[33] Jeong JS Kim HK Lee CR Park S Park JH Kang SW. Coexistence of chronic lymphocytic thyroiditis with papillary thyroid carcinoma: clinical manifestation and prognostic outcome. J Korean Med Sci. (2012) 27:883–9. doi: 10.3346/jkms.2012.27.8.883 PMC3410235 22876054

[34] Lim ES Shah SG Waterhouse M Akker S Drake W Plowman N. Impact of thyroiditis on 131I uptake during ablative therapy for differentiated thyroid cancer. Endocrine connections. (2019) 8:571–8. doi: 10.1530/EC-19-0053 PMC6499916 30965284

[35] De Leo S D'Elia S Grani G Dondi F Bertagna F Puxeddu E. A prospective multicenter study examining the relationship between thyroid cancer treatment outcomes and the presence of autoimmune thyroiditis. Thyroid. (2023) 33:1318–26. doi: 10.1089/thy.2023.0052 37725571

